# Benzodiazepines prescribing in eldery patients: A study about the prescribing behaviour of tunisian psychiatrists

**DOI:** 10.1192/j.eurpsy.2021.1142

**Published:** 2021-08-13

**Authors:** M. Lagha, U. Ouali, F. Nacef

**Affiliations:** Department Of Psychiatry A, Razi hospital, Manouba, Tunisia

**Keywords:** Benzodiazepines, Prescribing, psychiatry, eldery

## Abstract

**Introduction:**

Prescribing benzodiazepines (BZD) in patients over 65 years old requires special precautions in view of the frequency and the severity of their side effects, especially in this age group.

**Objectives:**

The objectives of our work were to evaluate the modalities of BZDs prescribing in elderly patients in psychiatry and to assess their compliance with international recommendations.

**Methods:**

This is a descriptive cross-sectional study conducted through a Google-forms self-administered questionnaire, intended for psychiatrists and psychiatric residents, over a period of two months, from April 1 to May 31, 2019.

**Results:**

One hundred physicians practicing in psychiatry answered our questionnaire. The response rate was 28%. Special precautions were taken in elderly patients by 96.5% of the participants. In eldery patients, long half-life BZDs were prescribed in 15% of cases. The majority of the participants indicated that the risk of falls (98.1%) and memory impairments (75.2%) were the main risks to which they were particularly vigilant during the prescribing of BZDs in eldery patients. In the elderly, 20% of the participants said they did not take special precautions when stopping BZDs.

**Conclusions:**

The frequency and severity of side effects associated with BZDs in the elderly are the cause of strict prescribing rules in this age group. According to the results of our study and to the literature data, the prescribing practices of these molecules in the elderly remain insufficiently in accordance with the guidelines.

